# Discriminating Breast Cancer Patients by Spectral Analysis of Arterial Pulse Waveforms

**DOI:** 10.1007/s13239-026-00842-0

**Published:** 2026-05-18

**Authors:** Chao-Tsung Chen, Ting-Wei Yeh, Yi-Sheng Chou, Chi-Feng Cheng, Yi-Hsuan Lai, Chung-Hua Hsu, Hsin Hsiu

**Affiliations:** 1 Institute of Traditional Medicine, College of Medicine, National Yang-Ming Chiao-Tung University, Taipei, Taiwan; 2 Department of Traditional Chinese Medicine, Taipei City Hospital RenAi Branch, Taipei, Taiwan; 3 Department of Recreation and Sport Management, University of Taipei, Taipei, Taiwan; 4 Graduate Institute of Biomedical Engineering, National Taiwan University of Science and Technology, No.43, Section 4, Keelung Road, Taipei, Taiwan; 5 Division of Hematology and Oncology, Department of Medicine, Taipei City Hospital, Renai Branch, Taipei, Taiwan; 6 School of Chinese Medicine, College of Medicine, National Yang-Ming Chiao-Tung University, 155, Li-Nong St, Sec 2, Peitou, Taipei, Taiwan; 7 Department of traditional Chinese medicine, National Yang-Ming Chiao-Tung University Hospital, Yilan, Taiwan; 8 Biomedical Engineering Research Center, National Defense Medical University, Taipei, Taiwan

**Keywords:** Breast cancer, Pulse, Spectral analysis, Noninvasive screening, Machine learning

## Abstract

**Background/Aim:**

Breast cancer (BC) remains a major health threat, highlighting the need for accessible screening. Characteristics of the cardiovascular system can be altered in BC patients. This study explored a noninvasive method combining arterial pulse-waveform analysis and classification to compare arterial pulse-waveform characteristics between BC patients (*n* = 51)) and age-matched controls (*n* = 76)).

**Methods:**

From one-minute radial waveform recordings, 40 harmonic indices (amplitude proportions, phase angles, and their variability) were derived. Significant differences in pulse-waveform indices were observed between groups, suggesting altered pulse-wave transmission conditions in BC patients. Two classifiers were compared: machine learning and a novel pulse-distribution analysis (PDA).

**Results:**

PDA demonstrated an acceptable discrimination with an AUC of 0.74, outperforming machine learning (AUC up to 0.58). Subgroup analysis by clinical factors such as TNM stage showed variable performance, with AUCs ranging from 0.66-0.93. Discrimination appeared to improve with disease progression (AUC 0.86 for stage IV), suggesting that tumor-induced vascular alterations may affect pulse-wave transmission.

**Conclusion:**

The primary contribution of this work is the identification of distinct pulse-waveform signatures in BC patients, providing a physiological basis for future development of noninvasive screening tools. The proposed method, characterized by its low cost and ease of use, warrants further validation in larger cohorts.

**Supplementary Information:**

The online version contains supplementary material available at https://doi.org/10.1007/s13239-026-00842-0.

## Introduction

Breast cancer (BC) seriously endangers the health of women due to it being one of the leading causes of cancer-associated death. The early diagnosis of different types of cancer has become a necessity in cancer research, including since this can facilitate the subsequent clinical management of patients and also aid prognosis predictions [1]. The early identification of BC can markedly improve the efficacy of applied treatments and therefore be hugely beneficial to women’s health [1–4].

At present, the most commonly used methods for the early detection of BC in clinical practice include breast self-examination, breast ultrasound, and mammography [1, 3, 5–8]. In addition, precision genetic testing of the status of hormone receptors such as the estrogen receptor (ER), progesterone receptor (PR), and human epidermal growth factor receptor 2 (HER2) has also been used to classify BC phenotypes and help in the selection of treatment modalities. Many studies reported in the literature have applied other strategies for enabling early diagnosis and prognosis prediction for cancer. For example, profiling of circulating miRNAs has been demonstrated to be a promising method for cancer detection and identification, although it suffers from low sensitivity for screening in the early stages and from difficulty in discriminating benign from malignant tumors. The utilization of gene signatures could significantly improve the ability to determine the prognosis in cancer patients [1, 4, 11–13].

An important recent trend has been to combine classification technology to further improve the detection accuracy. Classification is a learning process that categorizes data into a set of finite classes. Different machine-learning (ML) techniques and feature-selection algorithms have been widely applied to disease prognosis prediction; these techniques have been used to model the progression and treatment effects in cancerous conditions, and can be used to find certain types of cancer before they cause symptoms. The application of classification techniques has significantly improved the accuracy of cancer prediction outcomes by 15–20% [1, 4].

The ability of classification techniques to detect key features in complex datasets can facilitate beneficial decision-making. Most predictions in cancer applications have been made by integrating genomic, clinical, histological, imaging, demographic, epidemiological, and proteomic data. Commonly used input features include gene expression profiles, clinical variables, and histological parameters. The family history, age, diet, weight, high-risk habits, and exposure to environmental carcinogens may also play critical roles in predicting the development of cancer [3, 4, 10, 11, 14–16]. In addition, molecular biomarkers, cellular parameters, and the expression of certain genes have been demonstrated to be informative for cancer prediction [1].

Blood flow is an important factor affecting the metabolism and oxygenation of tumor tissue [17]. Imaging methods such as PET, MRI, and Doppler ultrasound can be used to monitor blood-flow responses, and have been proposed for playing important roles in cancer management, including for screening, diagnosis, and disease staging [17, 18]. Vessel properties can also change during cancer progression. Tumor blood vessels have been reported to exhibit abnormal morphology [19]; they may have irregular diameters and can be fragile and leaky, and hence can be characterized by abnormal blood flow [20]. The induced changes in blood flow may alter the shape, size, and differentiation of the endothelium, possibly via the aberrant expression of flow-mediated transcription factors [21]. These structural changes can include negative arterial remodeling—which can induce changes in the aortic stiffness such as decreased distensibility—being linked to an increased risk of cardiovascular events [22].

Pulse-waveform analysis can be used to assess changes in the elastic properties of blood vessels, since pulse waves are transmitted through the main arteries propelled by the heart beating. Time-domain pulse analysis has been widely investigated for the detection of vascular aging and cardiovascular disease [23–25]. The interaction between the mechanical properties of the elastic vessel walls and internal dynamics of blood flow can be crucial for developing effective clinical intervention strategies [26]. Changes in vascular elasticity may be associated with hyperlipidemia, and this mechanism has been applied to the development of arteriosclerosis therapy [27]. Frequency-domain pulse analysis may help reduce the effects of small waveform perturbations on the results of such analyses. In combination with classification techniques, spectral analysis has been used to explore the feasibility of detecting and evaluating different diseases and physiological states by identifying subtle changes in vascular characteristics, such as vascular aging, Alzheimer’s disease, sarcopenia, side effects of COVID-19 vaccines, and different running levels [28–32]. These applications suggest that measuring and analyzing the pulse waveform to monitor the arterial pulse-wave transmission condition could be useful for evaluating the effects of cancer tissues on the cardiovascular system.

A basic requirement when using classification techniques to model a disease is for the training dataset to be sufficiently large [1]. However, in practical clinical studies it is often difficult to obtain a sufficiently large sample for classification analysis. To address this problem, we have worked on developing an alternative classification method called pulse-distribution analysis (PDA) that can preliminarily and effectively assess the efficacy of classification for a smaller number of subjects. This PDA classification technique has been applied to investigating changes in the pulse waveform induced by diseases or physiological states, and its classification performance (quantified as the AUC) has been found to be similar to that of ML analysis [30, 32].

We have previously used frequency-domain analysis to detect significant differences in spectral pulse indices between BC (or colorectal cancer) patients and control subjects, and found that different cancers may show different patterns of spectral indices [33–35]. However, the numbers of participants in these previous studies were insufficient for using classification analysis to further explore the feasibility of screening subjects with specific types of cancer. The present study advances existing research by using noninvasive pulse-waveform analysis with spectral and two classification techniques (PDA and ML; based on the independent classification of each model) to discriminate BC patients from controls. Since the basic assumption underlying the use of pulse waves for BC detection is that tumor tissue may cause changes in pulse-wave transmission, several factors that may affect pulse-wave transmission—age, the three-highs (high blood pressure, high cholesterol, and high blood sugar), and TNM stage—were also a focus of the present study.

This study investigated an integrated approach that combines noninvasive arterial pulse sensing with spectral and classification analysis. Based on the observation that tumor progression may influence systemic vascular properties, we examined whether these changes could be captured through harmonic variations in the pulse waveform. A custom pulse-distribution analysis (PDA) was compared against ML methods using data from 51 patients and 76 controls. While demonstrating feasibility for BC screening, this cost-effective technique shows broader potential as a noninvasive health monitoring tool worthy of further validation. The findings may offer insights into the relationship between vascular dynamics and oncological conditions, with possible implications for future noninvasive health monitoring strategies.

## Materials and Methods

The present experimental setup and the signal processing methods have been reported in detail previously [29–32]. The described work was carried out in accordance with The Code of Ethics of the World Medical Association (Declaration of Helsinki) for experiments involving humans.

The general and clinical characteristics of the study participants are listed in Table 1. The study population was recruited at the Division of Hematology and Oncology of the RenAi Branch of Taipei City Hospital. The study design was reviewed and approved by the institutional review board of Taipei City Hospital (approval number TCHIRB-10806006). Informed consent was obtained for experimentation with human subjects. The inclusion criteria were (1) aged > 20 years and (2) examinations performed in accordance with the NCCN Clinical Practice Guidelines in Oncology used to determine the presence of BC. The exclusion criteria were (1) severe comorbidity such as acute hepatitis, myocardial infarction, stroke, severe trauma, or other major surgeries within the previous 6 months; or (2) not suitable for enrollment as assessed by a physician.

**Table 1:** Characteristics of the subjects

	Control	BC	*p* value
Subject number	76	51	
Age (years)	59.65 ± 15.01	59.62 ± 11.58	0.99
HR (beats/min)	69.47 ± 11.43	70.32 ± 8.85	0.66
Hypertension	0	11	
Hyperlipidemia	0	3	
Cardiovascular disease	0	3	
Hypercholesterolemia	0	2	
Diabetes Mellitus	0	4	
TNM stage	0	51	
Stage 1	0	15	
Stage 2	0	19	
Stage 3	0	10	
Stage 4	0	7	

Before the measurements, the subjects were relaxed and rested for at least 10 min. All subjects gave their informed consent before experiments commenced, and did not consume food at least 1 h before each experiment. All subjects were non-smokers, and did not take coffee or drinks containing alcohol at least 1 day before experiments. For each experiment, the subjects were sitting on a chair, and 1-minute BPW signal was measured noninvasively. The BPW signal was acquired by a pressure transducer (KFG-2-120-D1-11, Kyowa) held onto the skin surface above the radial artery 2 cm from the left wrist. The signal was connected to a self-made converter circuit, and then connected to an analog-to-digital converter card (PCI- 9111DG, Adlink Technology, Taiwan) operating at a sampling rate of 1024 Hz. Before frequency-domain analysis, the signals were filtered using a digital 11th-order high-pass Chebyshev filter with a cutoff frequency of 0.01 Hz to eliminate baseline drift and reduce low-frequency interference (e.g., motion artifacts). Before the measurement, the heart rate (HR), brachial systolic BP and diastolic BP were measured by using a sphygmomanometer (MG150f, Rossmax). One thermistor was attached around the wrist to monitor the skin-surface temperature. The resistance of the thermistor was transformed into voltages that were also sampled every minute by the analog-to-digital converter card.

**Fig. 1:**
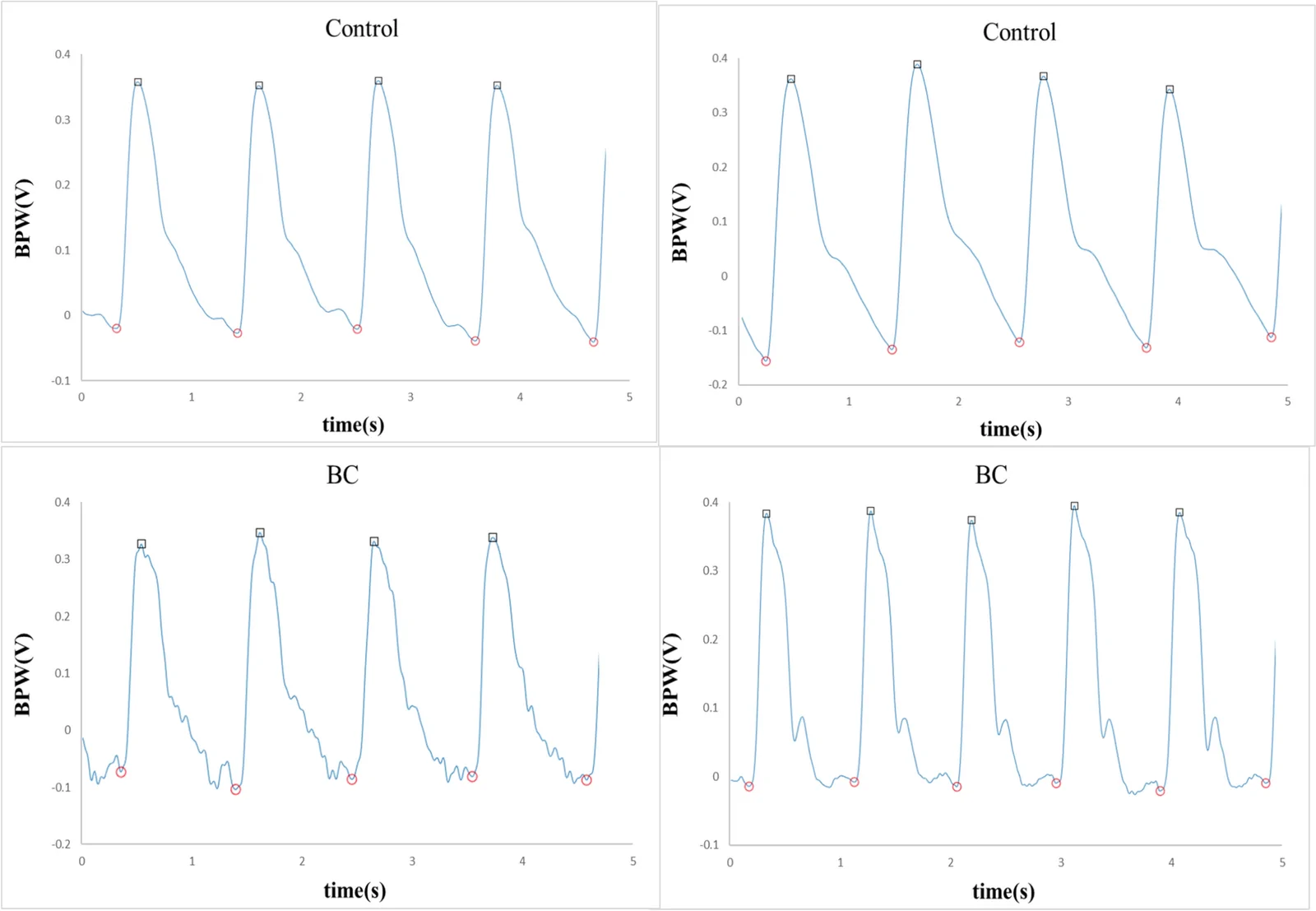
Typical measured BPW waveforms

The subjects were categorized into the BC and Control groups according to the professional judgment of the physician. One-minute radial blood-pressure waveform (BPW) signals were measured noninvasively (typical waveforms are shown in Fig. 1) between when BC was diagnosed and subsequent treatment (chemotherapy, radiotherapy, or surgery) was administered. Frequency-domain analysis was applied to derive harmonic indices from the measured BPW signals. Each individual pulse was segmented between consecutive foot points. Pulses were excluded if the value between the two foot points exceeded 20% of the pulse amplitude. Fourier analysis was then performed to decompose each pulse into harmonic components. For each harmonic *n* (*n* = 1–10), the amplitude (*Ampₙ*) and phase angle (*Pₙ*) were calculated as $Amp_{n} = \sqrt {A_{n}^{2} + B_{n}^{2} }$ and *Pₙ* = arctan(*Bₙ*/*Aₙ*), where *Aₙ* and *Bₙ* are the Fourier coefficients. The amplitude proportion Cₙ was defined as (*Ampₙ* / *Amp₀*) × 100%, where *Amp₀* is the DC component (mean value) of the pulse waveform. The coefficient of variation of *Cₙ* (*CVₙ*) and the standard deviation of *Pₙ* (*Pₙ_SD*) across all pulses in the 1-minute recording were also calculated to quantify pulse-to-pulse variability. Detailed procedures have been described previously [29–32]. Signal processing was performed using MATLAB (MathWorks); details of signal processing were described in the Supplemental Materials. Differences were assessed using two-tailed *t*-tests and were considered significant when *p* < 0.05; all *p* values were two-sided hypotheses.

For ML analysis, eight supervised classification methods were used to discriminate between BC and Control groups: support vector machine (SVM), multilayer perceptron (MLP), Gaussian naive Bayes (GNB), decision tree (DT), random forest (RF), logistic regression (LR), linear discriminant analysis (LDA), and K-nearest neighbors (KNN). Three-fold cross-validation was applied to evaluate model performance. The parameters of each ML model are provided in the Supplemental Materials.

For PDA, a feature scoring method was applied to classify subjects based on their spectral indices. First, spectral indices showing significant (*p* < 0.05) or marginally significant (0.05 < *p* < 0.10) differences between groups were selected. For each selected index, a subject’s value was converted into a score (0–10) based on its position within the distribution of the BC and Control groups. The final PDA score for each subject was calculated as the average of the individual scores from all selected indices. A threshold was then chosen to classify subjects into Control or BC groups. Detailed procedures have been described previously [30, 32] and are provided in the Supplemental Materials.

## Results

The spectral and classification analyses of arterial pulse waveforms yielded differences between breast cancer (BC) patients and age-matched controls.

Table 1 lists detailed characteristics of the subjects. The control group was free of major cardiovascular comorbidities, while the BC group included patients with conditions such as hypertension (*n* = 11) and cardiovascular disease (*n* = 3). Chi-square tests confirmed significant between-group differences in the prevalence of hypertension (*p* < 0.001) and other comorbidities.

### Harmonic Index Comparisons

**Fig. 2:**
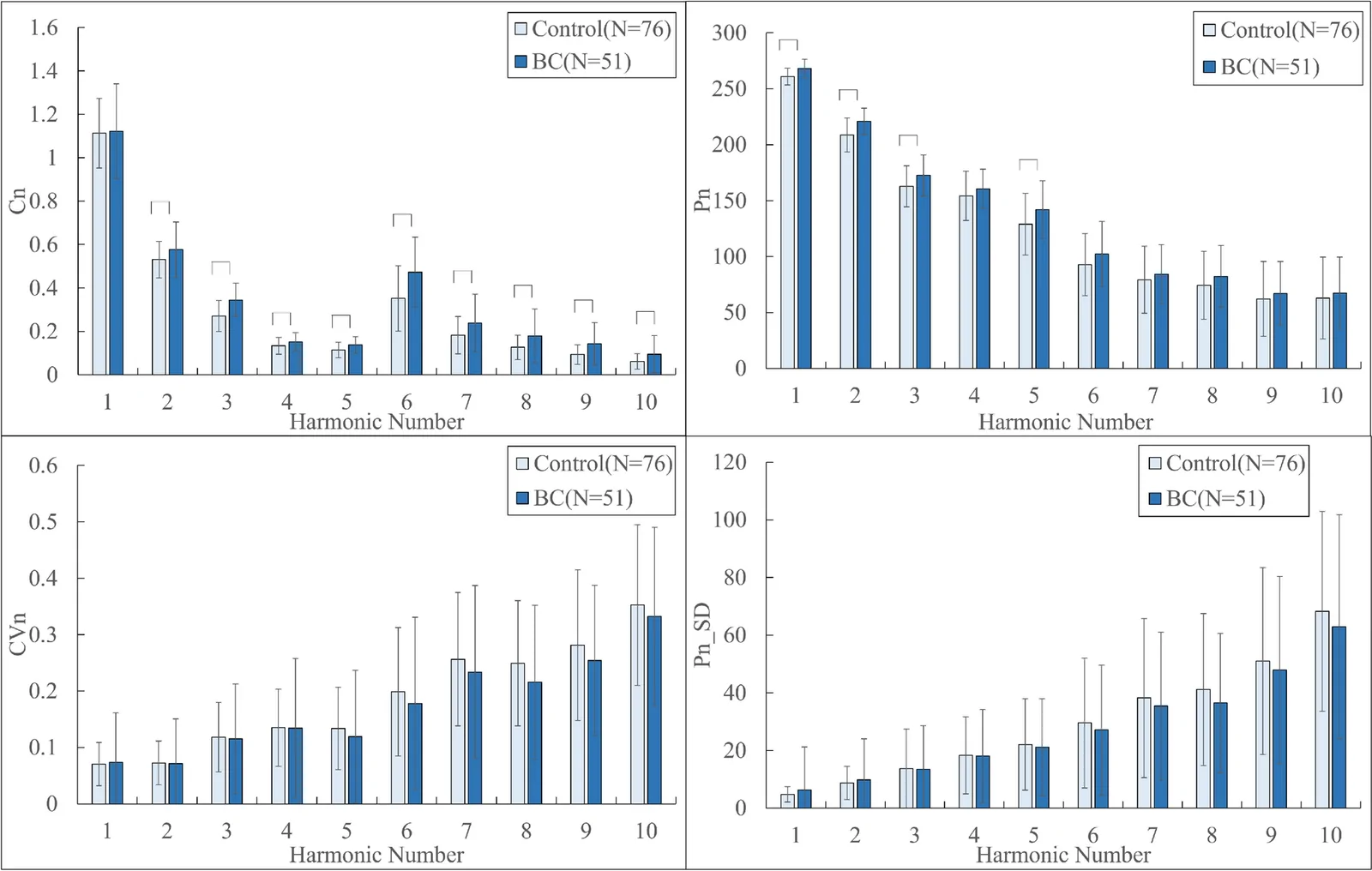
Comparisons of BPW harmonic indices between the BC and Control groups.Data are mean and SD values for *C*_*n*_, *CV*_*n*_, *P*_*n*_, and *P*_*n*__*SD*. *C*_5_–*C*_10_ values have been multiplied by 10 to make the differences clearer. “︹” indicates *p* < 0.05. *C*_*n*_ and *P*_*n*_ values for several harmonic components were significantly higher in the BC than the Control group

To establish the physiological basis for classification, we first compared the fundamental spectral harmonic indices between the BC and Control groups. As shown in Fig. 2, the BC group exhibited significantly higher amplitude proportions (*C*_*n*_) and higher phase angles (*P*_*n*_) for several harmonics compared to controls. These consistent differences across multiple frequency components indicate alterations in the pulse-wave transmission condition in BC patients, providing the foundational signal that subsequent classification analyses aim to capture.

### Classification Performance

Table 2 summarizes the performance of eight ML models in discriminating BC patients from controls using three-fold cross-validation. The linear discriminant analysis (LDA) classifier achieved the highest average AUC of 0.53 (95% CI not applicable due to cross-validation), which is only marginally above random chance (AUC = 0.50). Notably, no ML model demonstrated clinically useful discrimination, with the highest average AUC of 0.53 (LDA) and the highest average accuracy of 65.42% (SVM). The models also exhibited substantial instability and high variance across the three cross-validation folds, as evidenced by the fluctuating sensitivity and specificity values. For instance, the sensitivity of the Gaussian Naive Bayes (GNB) model ranged from 0.05 to 0.96. This consistent underperformance and instability across diverse ML algorithms suggest that the extracted pulse-waveform indices, in their current form, may not provide a sufficiently robust linear or non-linear signal for ML models to effectively learn the distinction between groups within this dataset.

**Table 2:** Results of the ML analyses comparing BPW indices between the BC and Control groups

BC vs Control								
Accuracy (%)	SVM	MLP	GNB	DT	RF	LR	LDA	KNN
1	73.15	64.06	69.06	55.98	66.31	69.02	64.55	59.27
2	68.57	67.22	32.04	59.62	65.24	68.33	67.01	60.63
3	54.56	53.38	44.95	50.98	49.39	52.34	64.30	52.34
Average	65.42*	61.55	48.68	55.52	60.31	63.23	65.28	57.41
Sensitivity								
1	0.01	0.18	0.05	0.37	0.13	0.03	0.03	0.21
2	0.00	0.13	0.96	0.29	0.15	0.03	0.17	0.28
3	0.01	0.31	0.86	0.39	0.17	0.14	0.55	0.33
Average	0.006	0.20	0.62*	0.35	0.15	0.06	0.25	0.27
Specificity								
1	0.98	0.80	0.91	0.62	0.84	0.92	0.85	0.72
2	1.00	0.91	0.02	0.73	0.87	0.98	0.89	0.75
3	0.93	0.69	0.14	0.59	0.73	0.80	0.70	0.66
Average	0.97*	0.80	0.35	0.64	0.81	0.90	0.81	0.71
AUC								
1	0.49	0.49	0.48	0.50	0.49	0.47	0.44	0.47
2	0.50	0.52	0.49	0.51	0.51	0.50	0.53	0.51
3	0.47	0.50	0.50	0.49	0.45	0.47	0.63	0.49
average	0.48	0.50	0.49	0.50	0.48	0.48	0.53*	0.49

Training and validation results are presented for threefold cross-validation. Asterisk indicates the best performance among the eight ML methods. For discriminating between BC and Control subjects, LDA had the highest AUC of 0.53

**Fig. 3:**
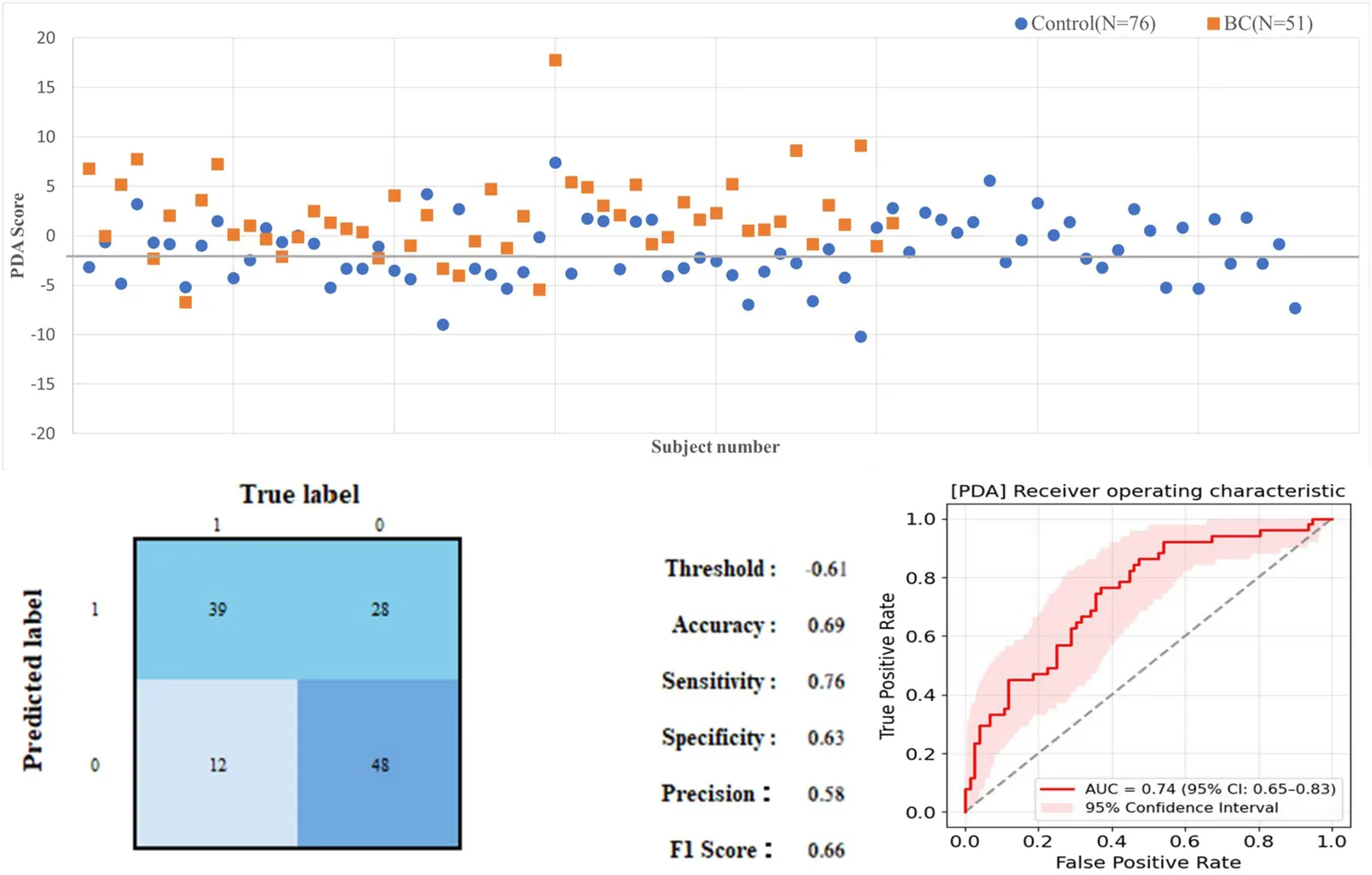
Distribution of PDA scores for BPW indices.Comparison between the BC and Control groups. Choosing – 0.61 as the score threshold resulted in AUC = 0.74 (95% CI: 0.65-0.83), precision = 0.58, and F1 score = 0.66. The PDA score were − 2.66 ± 3.35 and 1.94 ± 2.60 for Groups Control and BC, respectively (*p* < 0.01 by t-test). Under the high-specificity condition (specificity = 0.90), the corresponding sensitivity was 0.35

Having established fundamental differences in pulse characteristics, we next evaluated the ability of the PDA method to discriminate between the groups at the subject level. The distribution of PDA scores (Fig. 3) shows a clear separation between the BC and Control cohorts (*p* < 0.01). The PDA method achieved an AUC of 0.74 (95% CI: 0.65-0.83; with a precision of 0.58 and an F1 score of 0.66, outperforming all ML approaches listed in Table 2 with best ML AUC: 0.53) demonstrating that the subtle, population-level differences seen in Fig.2 can be integrated into a model with acceptable discrimination. Furthermore, under the high-specificity condition (0.90), PDA had a sensitivity of 0.35.

### Subgroup Analyses

**Fig. 4:**
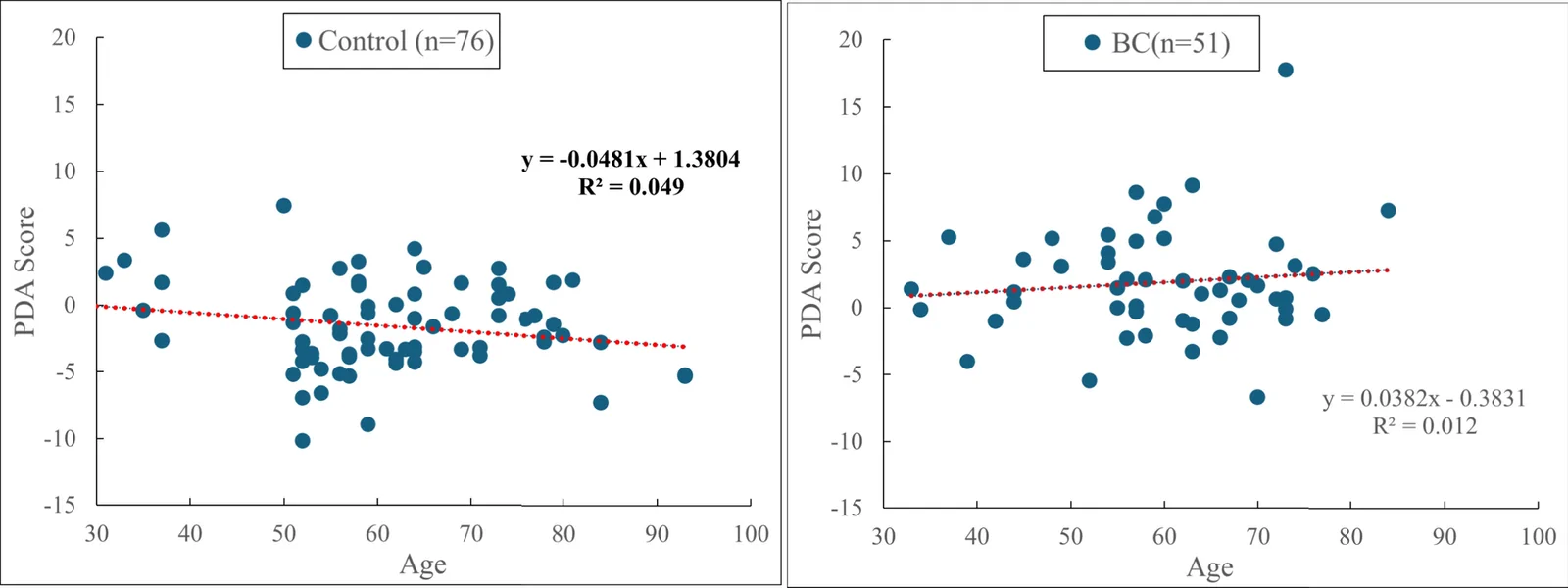
Correlations between PDA scores and age in all subjects. The *R*^2^ values for both groups were all < 0.05 (*p* > 0.1 in the *F*-test)

To strengthen the specificity of our findings for BC, we investigated age as a potential confounding factor, given its known strong correlation with vascular stiffness. As shown in Fig. 4, the correlation between PDA scores and age was negligible in both the BC (*R*^2^ < 0.05, *p* > 0.1) and Control groups. This lack of a strong linear relationship indicates that the discrimination power of the PDA score is primarily driven by factors other than age, most likely the pathological state of breast cancer.

The classification performance of pulse-wave analysis was further evaluated across clinically relevant subgroups. Table 3 presents detailed performance metrics across clinical subgroups and lists how the following factors affected the classification performance: TNM stage, tumor location, biomarkers, menopause status, and the three-highs. Table 3 indicates that the AUC values for discriminating between the Control and BC groups with these factors were between 0.66 and 0.93.

**Table 3:** Effects of various factors on the PDA classification performance

Age	Control, *n*	BC, *n*	AUC(95% CI)	Sensitivity(specificity = 0.90)	p-value (BC vs Control)	Precision	F1-score
< 50	10	10	0.86 (0.68-0.99)	0.50	0.002*	0.75	0.82
50 ~ 59	31	15	0.81 (0.67-0.93)	0.50	< 0.001*	0.58	0.65
60 ~ 69	16	14	0.88 (0.74-0.98)	0.57	< 0.001*	0.80	0.83
≥ 70	19	12	0.84 (0.67-0.97)	0.50	0.002*	0.69	0.79
TNM stage	Control, *n*	BC, *n*	AUC(95% CI)	Sensitivity(specificity = 0.90)	p-value (BC vs Control)	Precision	F1-score
1	76	15	0.79 (0.65-0.90)	0.40	< 0.001*	0.38	0.49
2	76	19	0.74 (0.59-0.86)	0.32	0.001*	0.46	0.53
3	76	10	0.86 (0.74-0.95)	0.53	< 0.001*	0.43	0.50
4	76	7	0.86 (0.66-0.99)	0.58	< 0.001*	0.42	0.53
Menopause	Control, *n*	BC, *n*	AUC(95% CI)	Sensitivity(specificity = 0.90)	p-value (BC vs Control)	Precision	F1-score
Yes	76	39	0.76 (0.67-0.85)	0.45	< 0.001*	0.53	0.64
No	76	12	0.85 (0.74-0.94)	0.42	0.001*	0.43	0.57
Biomarker	Control, *n*	BC, *n*	AUC(95% CI)	Sensitivity(specificity = 0.90)	p-value (BC vs Control)	Precision	F1-score
Tubular A	76	16	0.77 (0.63-0.89)	0.43	< 0.001*	0.36	0.49
Tubular B1	76	13	0.80 (0.65-0.91)	0.31	< 0.001*	0.32	0.47
Tubular B2	76	10	0.88 (0.77-0.96)	0.50	< 0.001*	0.38	0.52
HER2	76	9	0.78 (0.60-0.93)	0.44	< 0.001*	0.27	0.40
Triple negative	76	3	0.93 (0.85-1.00)	0.67	< 0.001*	0.23	0.38
Tumor Location	Control, *n*	BC, *n*	AUC(95% CI)	Sensitivity(specificity = 0.90)	p-value (BC vs Control)	Precision	F1-score
UIQ	76	10	0.81 (0.67-0.93)	0.40	0.002*	0.37	0.48
UOQ	76	21	0.82 (0.73-0.90)	0.38	< 0.001*	0.42	0.54
LIQ	76	6	0.79 (0.60-0.95)	0.33	0.018	0.27	0.38
LOQ	76	8	0.66 (0.45-0.84)	0.26	0.076	0.18	0.28
Three-highs	Control, *n*	BC, *n*	AUC(95% CI)	Sensitivity(specificity = 0.90)	p-value (BC vs Control)	Precision	F1-score
Yes	76	16	0.78 (0.65-0.90)	0.50	< 0.001*	0.38	0.50
No	76	35	0.73 (0.63-0.83)	0.26	< 0.001*	0.53	0.55

For the *p* values, those comparisons meeting the Bonferroni correction criterion (significance threshold was adjusted by dividing the conventional α level of 0.05 by the number of comparisons) were considered statistically significant and denoted as “*”.

Regarding age-specific performance, Fig. 4 shows the correlations between PDA scores and age in the BC and Control groups; the *R*^2^ values were all < 0.05 (*p* > 0.1 in the *F*-test). The BC detection abilities were determined when the subjects were divided into 10-year age groups, as indicated in Table 3. The AUC values in the age subgroups were all higher than that in Fig. 3 (0.74), being 0.86 (precision = 0.75; F1 score = 0.82) for those aged < 50 years, 0.88 for those aged 60-69 (precision = 0.80; F1 score = 0.83), and 0.84 for those aged ≥ 70 years (precision = 0.69; F1 score = 0.79). Notably, under the high-specificity condition (0.90), the sensitivity values in the four age subgroups (0.50–0.57) were all markedly higher than that in Fig. 3 (0.35).

Regarding disease stage stratification, for all TNM subgroups listed in Table 3, the AUC values were comparable to or higher than the overall performance (AUC = 0.74). The AUC value was highest for stages 3 and 4 (0.86 for both, indicating excellent discrimination; precision = 0.43 and 0.42; F1 score = 0.50 and 0.53, respectively) and lowest for stage 2 (0.74, indicating acceptable discrimination; precision = 0.46; F1 score = 0.53). Under the high-specificity condition (0.90), the sensitivity values in the four TNM subgroups (0.32–0.58) were all close to or higher than that in Fig. 3 (0.35), and highest in the stage−4 group (0.58). The method demonstrated progressively improved discrimination with advancing TNM stage (Stage I: AUC = 0.79; sensitivity 0.40 at specificity 0.90; Stage IV: AUC = 0.86; sensitivity 0.58 at specificity 0.90).

For the menopause subgroups, the AUC values were 0.76 (precision = 0.53; F1 score = 0.64) and 0.85 (precision = 0.43; F1 score = 0.57). Under the high-specificity condition (0.90), the sensitivity values in the menopause subgroups (0.45 and 0.42) were both higher than that in Fig. 3 (0.35). Both subgroups showed improved performance versus the overall cohort.

For the three-highs subgroups, the AUC values (0.78 with precision = 0.38 and F1 score = 0.50; 0.73 with precision = 0.53 and F1 score = 0.55) were both close to that in Fig. 3 (0.74). Under the high-specificity condition (0.9), the sensitivity values in the three-highs subgroups (0.50 and 0.26) were somewhat higher than or close to that in Fig. 3 (0.35). Patients with three-highs showed slightly better discrimination (AUC = 0.78) and achieved higher sensitivity (0.50 vs 0.26) at high specificity.

For the biomarker subgroups listed in Table 3, the AUC values were all somewhat higher than that in Fig. 3 (0.74). The AUC value was highest for tubular B2 (0.88) and triple negative (0.93, indicating outstanding discrimination; warrants further investigation in larger cohorts) and lowest for tubular A (0.77). Under the high-specificity condition (0.9), the sensitivity values in several biomarker subgroups were higher than that in Fig.3 (0.35), and were highest for tubular B2 (0.50) and triple negative (0.67).

For the tumor-location subgroups listed in Table 3, the AUC values were all slightly higher than or close to that in Fig.3 (0.74). The UOQ group accounted for the largest proportion of the BC subjects (21 of 51); the AUC value in the UOQ group was 0.82 (precision = 0.42 and F1 score = 0.54). Under the high-specificity condition (0.90), the sensitivity value in the UOQ group (0.38) was somewhat higher than that in Fig.3 (0.35).

## Discussion

### Comparison of Pulse Indices

The significant differences in harmonic indices observed in Fig.2 provide the crucial physiological rationale for our study. The elevated amplitude proportions and phase angles in BC patients are consistent with the hypothesis of increased arterial stiffness and altered wave transmission efficiency induced by the tumor’s systemic effects. Without these foundational differences, any attempt at classification would be unfounded. Thus, Fig.2 directly supports our conclusion that BC induces measurable vascular changes that can be probed via pulse-wave analysis.

This study used two types of pulse-waveform indices to evaluate the vascular effects in BC patients. Knowledge of the spectral distribution of the pulse waveform might facilitate the understanding of the pulse-wave transmission condition and thus evaluations of the blood-flow supply condition. Since most of the spectral power of the pulse waveform is concentrated in the first ten harmonic frequencies, the pulse indices of the first ten harmonics were selected to provide a better description of the pulse waveform. Pulse variability indices can be correlated with the regulatory activities influencing the perfusion condition of the blood supply. The differences in amplitudes and phase angles found between the groups in this study reflect changes in the pulse-wave transmission conditions induced by BC. The amplitude proportion can be affected by the propagation efficiency of pulse-wave transmission for specific harmonic components, which may in turn affect the blood-flow distribution. The phase angle corresponds to time delays in arterial pulse-wave transmission for different harmonic components. This can be correlated with the transmission speed of the arterial pulse, and can be affected by vascular elasticity, such as the stiffness of the main arteries or the compliance of local vascular beds.

As mentioned in the Introduction, the development of tumors may be accompanied by changes in vascular elasticity. It has been suggested that the risk of cancer is slightly but significantly increased in hypertensive patients [36]. Hypertension and antihypertensive drugs were noted to be able to influence cancer cells [37]. It has also been suggested that hypertension is related to the shortening of telomeres, leading to cell senescence and complications, including atherosclerosis and cancer [38]. It is highly feasible that an increased vascular stiffness in BC patients will increase the efficiency of pulse-wave transmission along the main arteries, and this was supported by the amplitudes of several frequency components in the pulse waveform becoming higher for BC patients, as revealed in Fig. 2.

The phase angle is another important spectral BPW index, with a higher phase angle representing leading in pulse-wave transmission. Fig.2 shows that the *P*_*n*_ values were higher for most of the analyzed frequency components (significantly for all except *P*_4_) in the BC subjects than in the Control subjects. It is highly feasible that a higher arterial stiffness results in the harmonic components within the BPW propagating along the main arteries more rapidly, hence leading to their higher *P*_*n*_ values.

### Classification Performance

Among the various tools used for the early detection of BC combined with classification technology, imaging methods are the most explored and they can achieve good detection results. For example, mammography-based diagnosis of BC through ML methods can achieve an AUC of 0.945 [3]. Evaluation tools based on digital mammograms for assessing the BC risk can achieve a detection AUC of 0.81 [8]. Analyzing radiomic features extracted from grayscale ultrasound B-mode images achieved an AUC of 0.824 [9]. An artificial neural network trained on a large dataset of consecutive mammography scans could discriminate between benign and malignant disease and accurately predict the probability of BC with an AUC of 0.965 [10]. Applying a deep-learning system to screening mammography data yielded an AUC of 0.82 in classifying invasive BC [6]. Analyzing the OPTIMAM Mammography Imaging Database made it possible to predict the risk of BC within the next 3 years with an AUC of 0.73 [5]. Combining surface-enhanced Raman spectroscopy with deep-learning techniques achieved an AUC of 0.9884 in detecting BC.

The above-described findings indicate that medical imaging methods can be excellent for detecting BC. However, various factors such as convenience and cost have resulted in diverse other technologies being widely explored to assist in the early screening of BC. Many studies have investigated the efficacy of analyzing various blood components in BC detection. For example, monitoring changes in T-cell receptors in peripheral blood has been suggested for the early detection of various cancers; using TCRβ sequences achieved an AUC of 0.86 in early-stage cancer detection [11]. Using single-nucleotide polymorphisms as key discriminators for BC in SVM classification achieved a predictive power of 69% [12]. Applying ML analysis to plasma extracellular vesicles as a noninvasive early diagnostic biomarker for BC in preclinical stages (mostly TNM stages 0–2) achieved an AUC of 0.82 [4], while using complete blood counts achieved an AUC of 0.64 in BC screening [14]. While each of these methods can achieve good detection performance, their cost is an important issue. This has prompted explorations of other approaches, including combining early-life body-size measures such as birthweight, child weight, height, and body mass index with a feedforward neural network, which achieved an AUC of 0.60 in detecting the BC risk [16].

The PDA model’s discrimination performance (Fig.3) is a direct consequence of the systematic pulse-wave alterations documented in Fig.2. By transforming the multi-dimensional harmonic differences into a single-dimensional PDA score, our method effectively summarizes the vascular properties associated with BC. The achieved AUC of 0.74, while moderate, confirms that the biological signal of interest is not merely statistically significant but also machine-detectable, bridging the gap between group-level physiology and subject-level classification. This performance was slightly better than the present ML analysis method, indicating that the PDA classification technique may be useful for preliminary assessments with smaller sample sizes.

The better performance of our novel PDA method over ML approaches (Table 2) warrants further discussion. The fact that a diverse set of algorithms, including both linear (e.g., LDA, LR) and non-linear models (e.g., SVM, MLP, RF), failed to achieve AUCs substantially above 0.53 suggests that the challenge lies not in the model selection itself, but likely in the nature of the feature space and the dataset size. Two non-mutually exclusive explanations are plausible. First, the limited sample size (*n* = 127) relative to the number of features (40 harmonic indices) likely led to overfitting and an inability of the ML models to generalize, a common issue in biomedical data analysis. Second, the subtle, population-level differences in pulse waveforms induced by BC may be better captured by the customized PDA method, which is specifically designed to model the distribution of pulse characteristics, rather than by generic ML classifiers seeking complex decision boundaries. The superior performance of PDA in this context highlights its potential utility for small-sample studies where traditional ML approaches struggle. Future studies with larger cohorts are needed to determine if ML models, with access to more data, can eventually outperform or complement the simpler PDA approach.

Comparing the BC classification performance between the present study and various existing technologies reveals that the present analysis method provides discrimination capability that may be suitable for certain screening contexts. While medical imaging methods typically achieve higher AUC values (around 0.90), they require hospital environments and incur higher implementation costs. The present method offers advantages of easy operation, short measurement time, and low execution cost. The present method can be used to achieve similar or even better classification performance than methods other than medical imaging. Moreover, the present technology also has the advantages of easy operation, short measurement time, and low execution cost. Early characterization of BC diagnoses has been suggested to help achieve better prognoses by prioritizing clinical referrals and facilitating early treatments [9]. Therefore, the present technology may potentially bridge the intermediate stage of extensive early screening and warning signs.

Methods for the early detection of cancer should have high specificity in order to avoid too many false-positive test results, since they may lead to unnecessary anxiety and follow-up procedures. Therefore, the sensitivity under the high-specificity condition is often used as another important indicator when evaluating the performance of methods for the early detection of BC. For example, the detection sensitivity for BC was 0.33 for the CancerSEEK test (which screens eight types of cancer) under the high-specificity condition. The median sensitivities of the CancerSEEK test were 0.78, 0.73, and 0.43 for cancer at stages III, II, and I, respectively [39]. The present analysis method exhibited a sensitivity of 0.35 in BC detection, which is close to the result of 0.33 stated above, while the cost of the present method involving measurements of pulse-wave transmission can be much lower than that of the CancerSEEK test, which was estimated to be around US$ 500.

A comparison for performance comparison of BC detection methods is listed in Table 4. The present pulse-wave analysis (AUC 0.74) achieves comparable performance to blood-based ML methods (AUC 0.64–0.82; [4, 14]) but falls short of imaging (AUC 0.945–0.988; [3, 15]). However, it offers unique advantages in cost and accessibility, requiring only a pressure transducer (vs. expensive assays or imaging). The low sensitivity at high specificity (0.35) reflects its current limitation for early-stage detection, whereas advanced techniques like SERS (sensitivity 0.95; [15]) excel but remain impractical for screening. Combining pulse-wave indices with routine blood parameters (e.g., CBC; [14]) could enhance accuracy while retaining low cost. Wearable integration may address the "last-mile" challenge in resource-limited settings, where mammography or SERS are unavailable.

**Table 4:** Performance comparison of breast cancer detection methods

Method	Study population (BC/Controls)	Key features used	AUC	Cost & accessibility	References
Pulse-Wave PDA	51/76	Harmonic indices	0.74	Very low; wearable-compatible	-
Mammography + DL	5,000 + cases	Radiomic features	0.945	High; requires imaging center	[3]
Plasma EVs + ML	120/120	Extracellular vesicle RNAs	0.82	Moderate; lab-dependent	[4]
TCRβ sequencing	100/100	T-cell receptor diversity	0.86	High; specialized assay	[11]
Complete blood count + ML	1,200/1,200	Routine blood parameters	0.64	Low; widely available	[14]
SERS + CNN	200/200	Serum Raman spectra	0.988	High; advanced equipment	[15]

*DL* deep learning, *EVs* extracellular vesicles, *TCRβ* T-cell receptor beta, *SERS* surface-enhanced Raman spectroscopy, *CNN* convolutional neural network.

Regarding the potential for early detection, our findings from early-stage patients provide preliminary but tangible evidence. The PDA method achieved an AUC of 0.79 and a sensitivity of 0.40 (at a specificity of 0.9) in discriminating TNM Stage I patients (*n* = 15) from controls (Table 3). While this performance is modest and not yet competitive with gold-standard imaging, it demonstrates that the vascular signal associated with breast cancer is present and detectable even at early stages. Furthermore, in subjects under 50 years of age—a key demographic for early-onset cancer—the method achieved an AUC of 0.86. This suggests that the technique is not solely reliant on age-related vascular changes and may have value in younger populations.

Consequently, the proposed technique should not be viewed as a direct alternative to blood screening or imaging, but rather as a complementary pre-screening or risk-stratification tool. Its value proposition lies in its ultra-low cost, non-invasiveness, and rapid measurement time, making it suitable for large-scale population screening or longitudinal self-monitoring in high-risk groups. A practical application could be to identify a subset of individuals from a large, asymptomatic population who exhibit anomalous pulse-wave patterns, thereby prioritizing them for more specific and costly diagnostic investigations (e.g., mammography or liquid biopsy). This triage approach could improve the cost-effectiveness of existing screening programs.

### Possible Confounding Factors

This study used pulse-waveform analysis to evaluate the changes in the elastic properties of blood vessels induced by BC. Certain confounding factors that affect the vascular elastic properties can also affect the analyzed pulse spectral indices and thus the classification performance, and so the impact of these factors needs to be explored and understood. This present study explored the following possible confounding factors: age and the three-highs. It is also necessary to understand whether detailed characteristics of BC—such as the tumor location, TNM stage, and biomarkers—will affect the classification performance based on these pulse indices. The possible impact can be investigated using regression correlation analysis, PDA scores, and classification performance parameters such as the AUC. Although the numbers of subjects decreased after subgrouping, this part of the analysis was still instructive for future applications. Another important consideration is that PDA requires fewer subjects, and so it can provide reference trends for classification when there are fewer subjects after dividing the entire groups into subgroups.

#### Age

The arterial stiffness and systolic blood pressure can increase during aging; the pathogenesis of vascular aging has been attributed to oxidative stress, production of free radicals, and neuroendocrine and genetic changes [40, 41]. Regarding the confounding effect of age, the regression analysis results in Fig.4 show that the *R*^2^ values were < 0.05 in both the BC and Control groups, which indicates that age did not have prominent effect on the distribution of PDA scores.

Regarding the effects on the classification performance, Table 3 indicates that the AUC values in all age subgroups (0.81–0.88) were all higher than that of the entire BC group (0.74). Under the condition of high specificity, the sensitivity values were higher in all four age subgroups (0.50–0.57) than in the entire BC group (0.35), indicating that age grouping markedly improved the classification performance. This may have been due to the age concentration induced by grouping reducing confounding by vascular aging effects.

#### Menopause Status

Another age-related factor is the possible effect of menopause, since female hormone concentrations may affect the elastic properties of blood vessels. We therefore divided participants into two subgroups according to whether or not they had gone through menopause, and compared their detection AUC values with that in the Control group. Table 3 indicates that the AUC values (0.76 and 0.85) were somewhat higher than that in the entire BC group (0.74). There was greater improvement in sensitivity under the high-specificity condition, with the sensitivity values both being higher (0.45 and 0.42) than that in the entire BC group (0.35).

#### TNM Stage

TNM classification is a globally recognized standard for staging many types of malignant tumor on an anatomical scale. Previous studies have used the miRNA signature and transcriptome analysis to predict the stage of BC [42, 43]. For the present study, Table 3 indicates that the AUC values for all TNM-staging groups were all close to or higher (0.74–0.86, with 0.86 indicating excellent discrimination) than that in the entire BC group (0.74).

It was also found that this analysis method can provide better detection performance as the cancer stage increases. As indicated in Table 3, the AUC for subjects from stages 1 to 4 increased from 0.79 to 0.86. The sensitivity values under the high-specificity condition were also markedly higher for some TNM-stage subgroups (up to 0.58 for stage 4) than in the entire BC group (0.35). In addition, even for stage−1 BC subjects, this technique can achieve a sensitivity of 0.40 under the high-specificity condition, while it increased to 0.58 for stage 4. These findings indicate that the present technology has the potential to be applied to the auxiliary monitoring of the BC disease course as more cases are accumulated in the future.

#### Biomarkers

Tumor markers are bioactive substances secreted by tumors themselves or by the patient’s body in response to the presence of tumors, which can be used for tumor evaluations. Different tumors have different tumor markers, and the blood concentrations of tumor markers are positively correlated with the tumor stage. The most commonly used tumor markers for BC detection are hormone receptors (ER, PR, and HER2) and Ki67, which can provide important supplemental information in TNM anatomical staging. The expressed receptors can also be used when deciding about the choice of treatment drugs.

Table 3 indicates that the AUC values in the biomarker subgroups (0.77–0.93) were all higher than that in the entire BC group (0.74), including being ≥ 0.88 for tubular B2 and triple negative (although only involving three BC patients). The sensitivity values under the high-specificity condition for several biomarker subgroups were also higher than that in the entire BC group (0.35), with notable values of 0.50 for tubular B2 and 0.67 for triple negative. These results indicate that biomarker grouping can improve the classification efficiency.

#### Tumor Location

Tumors at different sites may exert different effects on pulse-wave transmission, which may also affect the classification performance of pulse-waveform indices. Therefore, in the present study we divided the subjects into four subgroups according to the tumor location to observe their effect on pulse-waveform indices.

Table 3 indicates that the AUC values for all location subgroups were close to or higher than that in the entire BC group, while the sensitivity values under the high-specificity condition were higher than that in the entire BC group. For the UOQ subgroup, which accounted for the largest proportion of the present BC subjects and also had the highest incidence rate in general clinical observations, the AUC value (0.82) was higher than that in the entire BC group (0.74). The sensitivity value (0.38) under the high-specificity condition was higher than that in the entire BC group (0.35).

#### The Three-highs

The three-highs can affect vascular elasticity and cause significant harm to affected patients [44]. For example, a high blood sugar concentration has been noted to increase the risk of arterial stiffness [45]. Table 3 indicates that the BC subjects could be separated according to whether or not they had the three-highs. It was found that when discriminated with the Control group, the AUC values (0.78 and 0.73) were both similar to that in the entire BC group (0.74) for subjects with and without the three-highs. The sensitivity values under the high-specificity condition were higher than or close to that in the entire BC group (0.35). Of particular note is that for BC subjects with at least one of the three-highs, the sensitivity value under the high-specificity condition improved to 0.50.

The absence of a significant correlation between PDA scores and age (Fig. 4) is a critical finding that bolsters the validity of our conclusions. It argues against the possibility that the group differences in Fig. 2 and the classification results in Fig. 3 are merely proxies for vascular aging. This allows us to state with greater confidence that the pulse-waveform signal captured by our method is more specifically associated with the presence of BC.

### Possible Contributions of Pulse-Wave Transmission Monitoring to BC Detection

This study found significant differences in pulse-waveform indices between the BC and Control groups, indicating that monitoring the pulse-wave transmission condition can assist in BC detection. The performance in discriminating between the entire BC group and the Control group achieved an AUC of 0.74 (indicating acceptable discrimination), with a sensitivity under the high-specificity condition of 0.35.

It was observed that the detection performance could be further increased by subdividing the subjects according to their condition, although the numbers of subjects in the different subgroups were small and hence the findings might be unreliable. The AUC values were somewhat higher in most subgroups than the overall value of 0.74 (in some subgroups it even reached > 0.80), and the sensitivity values under the high-specificity condition also showed an improvement trend in most subgroups (in 15 of 21 subgroups it was > 0.40). For example, the AUC and sensitivity values under the high-specificity condition improved to 0.86 and 0.58, respectively, for TNM stage 4, to 0.88 and 0.57 for those aged 60–69 years, and to 0.88 and 0.50 for the tubular-B2 biomarker. This indicates that the detection capabilities of the present method of pulse-waveform analysis may improve as BC progresses. The present results have also illustrated that our method has the potential to provide warning signs in younger patients (e.g., under 50 years old: AUC of 0.86, and a sensitivity of 0.50 under the high-specificity condition) and those with early-stage BC (e.g., TNM stage 1: AUC of 0.79, and a sensitivity of 0.40 under the high-specificity condition).

## Conclusion and Limitations

This study identified measurable differences in arterial pulse-wave characteristics between BC patients and controls through spectral harmonic analysis. The observed alterations in amplitude proportions and phase angles suggest changes in vascular properties associated with BC. The classification analysis using PDA demonstrated the detectability of these physiological differences, achieving an AUC of 0.74. This performance level indicates potential for further investigation but requires substantial improvement for clinical application.

The imbalance in comorbidities between groups represents a study limitation. Conditions like hypertension are known to affect arterial stiffness and pulse-wave characteristics. While this may contribute to the observed differences in pulse-wave indices, subgroup analysis of BC patients with at least one "three-highs" condition showed maintained discrimination capability (AUC = 0.78). This suggests the pulse-wave signal retains discriminatory power in populations with metabolic-vascular comorbidities. Nevertheless, it remains possible that the observed pulse-wave differences between groups are partially attributable to the higher prevalence of hypertension and cardiovascular disease in the BC group, rather than solely to the presence of the tumor itself. Future studies with matched designs (e.g., BC patients and non-cancer controls with similar cardiovascular risk profiles) are needed to isolate cancer-specific effects.

Additionally, based on the current study design (BC patients vs. healthy controls; we did not include other cancer types, and we cannot definitively rule out the presence of undiagnosed concurrent malignancies in the BC patients), we cannot determine whether the observed pulse-wave changes are specific to BC or may be shared with other cancer types. Future studies that include multiple cancer type groups under the same protocol, with confirmed single-cancer diagnoses, are needed to explore whether distinct vascular signatures exist for different malignancies.

This study has several limitations that should also be addressed in future work. The sample size, while sufficient for this preliminary investigation, remains modest and necessitates validation in larger, more diverse cohorts. The comparison between the PDA method and ML algorithms, while informative, may not fully represent the performance of optimally tuned ML models. Additionally, potential confounding effects from cardiovascular conditions, although partially addressed through subgroup analyses, warrant more systematic investigation in future studies with dedicated design.

To translate this approach into clinical utility, future work should focus on expanding datasets, standardizing measurement protocols, and exploring integration with other noninvasive biomarkers or wearable technologies. While not replacing current gold-standard techniques, this pulse-wave analysis method offers a complementary, accessible strategy to broaden early detection efforts, particularly in resource-limited settings or for longitudinal monitoring of high-risk populations.

## Supplementary Information

Below is the link to the electronic supplementary material.

Supplementary file1 (DOC 75 KB)

## Data Availability

The data cannot be made publicly available upon publication because they contain sensitive personal information. The data that support the findings of this study are available upon reasonable request from the authors.

## References

[1] Kourou, K., T. P. Exarchos, K. P. Exarchos, M. V. Karamouzis, and D. I. Fotiadis. Machine learning applications in cancer prognosis and prediction. *Comput. Struct. Biotechnol. J.* 15(13):8–17, 2014. 10.1016/j.csbj.2014.11.005 PMC4348437 25750696

[2] Del Corso, G., D. Germanese, C. Caudai, G. Anastasi, P. Belli, A. Formica, A. Nicolucci, S. Palma, M. A. Pascali, S. Pieroni, C. Trombadori, S. Colantonio, M. Franchini, and S. Molinaro. Adaptive machine learning approach for importance evaluation of multimodal breast cancer radiomic features. *J. Imaging. Inf. Med.* 2024. 10.1007/s10278-024-01064-3. PMC11300750 38478187

[3] Liu, J., J. Lei, Y. Ou, Y. Zhao, X. Tuo, B. Zhang, and M. Shen. Mammography diagnosis of breast cancer screening through machine learning: a systematic review and meta-analysis. *Clin. Exp. Med.* 23(6):2341–2356, 2023. 10.1007/s10238-022-00895-0 36242643

[4] Lin, L., G. X. Cai, X. M. Zhai, X. X. Yang, M. Li, K. Li, C. L. Zhou, T. C. Liu, B. W. Han, Z. J. Liu, M. Q. Chen, G. L. Ye, Y. S. Wu, and Z. W. Guo. Plasma-derived extracellular vesicles circular RNAs serve as biomarkers for breast cancer diagnosis. *Front. Oncol.* 10(11):752651, 2021. 10.3389/fonc.2021.752651 PMC8660094 34900700

[5] Ellis, S., S. Gomes, M. Trumble, M. D. Halling-Brown, K. C. Young, N. S. Chaudhry, P. Harris, and L. M. Warren. Deep learning for breast cancer risk prediction: application to a large representative UK screening cohort. *Radiol. Artif. Intell.* 6(4):e230431, 2024. 10.1148/ryai.230431 PMC11294956 38775671

[6] Condon, J. J. J., V. Trinh, K. A. Hall, M. Reintals, A. S. Holmes, L. Oakden-Rayner, and L. J. Palmer. Impact of transfer learning using local data on performance of a deep learning model for screening mammography. *Radiol. Artif. Intell.* 6(4):e230383, 2024. 10.1148/ryai.230383 PMC11294949 38717291

[7] Yasar, S., F. H. Yagin, R. Melekoglu, and L. P. Ardigò. Integrating proteomics and explainable artificial intelligence: a comprehensive analysis of protein biomarkers for endometrial cancer diagnosis and prognosis. *Front. Mol. Biosci.* 11:1389325, 2024. 10.3389/fmolb.2024.1389325 PMC11184912 38894711

[8] Santeramo, R., C. Damiani, J. Wei, G. Montana, and A. R. Brentnall. Are better AI algorithms for breast cancer detection also better at predicting risk? A paired case-control study. *Breast. Cancer. Res.* 26(1):25, 2024. 10.1186/s13058-024-01775-z PMC10848404 38326868

[9] Ferre, R., J. Elst, S. Senthilnathan, A. Lagree, S. Tabbarah, F. I. Lu, A. Sadeghi-Naini, W. T. Tran, and B. Curpen. Machine learning analysis of breast ultrasound to classify triple negative and HER2+ breast cancer subtypes. *Breast. Dis.* 42(1):59–66, 2023. 10.3233/BD-220018 36911927

[10] Ayer, T., O. Alagoz, J. Chhatwal, J. W. Shavlik, C. E. Kahn Jr., and E. S. Burnside. Breast cancer risk estimation with artificial neural networks revisited: discrimination and calibration. *Cancer.* 116(14):3310–3321, 2010. 10.1002/cncr.25081 PMC2920215 20564067

[11] Cai, Y., M. Luo, W. Yang, C. Xu, P. Wang, G. Xue, X. Jin, R. Cheng, J. Que, W. Zhou, B. Pang, S. Xu, Y. Li, Q. Jiang, and Z. Xu. The deep learning framework iCanTCR enables early cancer detection using the t-cell receptor repertoire in peripheral blood. *Cancer. Res.* 84(11):1915–1928, 2024. 10.1158/0008-5472.CAN-23-0860 38536129

[12] Listgarten, J., S. Damaraju, B. Poulin, L. Cook, J. Dufour, A. Driga, J. Mackey, D. Wishart, R. Greiner, and B. Zanke. Predictive models for breast cancer susceptibility from multiple single nucleotide polymorphisms. *Clin. Cancer. Res.* 10(8):2725–2737, 2004. 10.1158/1078-0432.ccr-1115-03 15102677

[13] Stojadinovic, A., A. Nissan, J. Eberhardt, T. C. Chua, J. O. Pelz, and J. Esquivel. Development of a Bayesian belief network model for personalized prognostic risk assessment in colon carcinomatosis. *Am. Surg.* 77(2):221–230, 2011. 21337884

[14] Araujo, D. C., B. A. Rocha, K. B. Gomes, D. N. da Silva, V. M. Ribeiro, M. A. Kohara, F. Tostes Marana, R. A. Bitar, A. A. Veloso, M. C. Pintao, F. H. da Silva, C. F. Viana, P. H. A. de Souza, and I. D. C. G. da Silva. Unlocking the complete blood count as a risk stratification tool for breast cancer using machine learning: a large scale retrospective study. *Sci. Rep.* 14(1):10841, 2024. 10.1038/s41598-024-61215-y PMC11089041 38736010

[15] Cheng, N., Y. Gao, S. Ju, X. Kong, J. Lyu, L. Hou, L. Jin, and B. Shen. Serum analysis based on SERS combined with 2D convolutional neural network and Gramian angular field for breast cancer screening. *Spectrochim. Acta. A. Mol. Biomol. Spectrosc.* 312:124054, 2024. 10.1016/j.saa.2024.124054 38382221

[16] Svendsen, S. M. S., D. C. Pedersen, B. W. Jensen, J. Aarestrup, L. Mellemkjær, L. G. Bjerregaard, and J. L. Baker. Early life body size and puberty markers as predictors of breast cancer risk later in life: a neural network analysis. *PLoS. One.* 19(2):e0296835, 2024. 10.1371/journal.pone.0296835 PMC10857724 38335218

[17] Choe, R., M. E. Putt, P. M. Carlile, T. Durduran, J. M. Giammarco, D. R. Busch, K. W. Jung, B. J. Czerniecki, J. Tchou, M. D. Feldman, C. Mies, M. A. Rosen, M. D. Schnall, A. DeMichele, and A. G. Yodh. Optically measured microvascular blood flow contrast of malignant breast tumors. *PLoS. One.* 9(6):e99683, 2014. 10.1371/journal.pone.0099683 PMC4072684 24967878

[18] McLaughlin, R., and N. Hylton. MRI in breast cancer therapy monitoring. *NMR. Biomed.* 24(6):712–720, 2011. 10.1002/nbm.1739 PMC4509744 21692116

[19] Warren, B. A., P. Shubik, R. Wilson, H. Garcia, and R. Feldman. The microcirculation in two transplantable melanomas of the hamster. I. in vivo observations in transparent chambers. *Cancer. Lett.* 4:109–116, 1978. 10.1016/s0304-3835(78)93797-7 647650

[20] Dudley, A. C. Tumor endothelial cells. *Cold. Spring. Harb. Perspect. Med.* 2(3):a006536, 2012. 10.1101/cshperspect.a006536 PMC3282494 22393533

[21] De Val, S., and B. L. Black. Transcriptional control of endothelial cell development. *Dev. Cell.* 16:180–195, 2009. 10.1016/j.devcel.2009.01.014 PMC2728550 19217421

[22] Grover, S., P. W. Lou, C. Bradbrook, K. Cheong, D. Kotasek, D. P. Leong, B. Koczwara, and J. B. Selvanayagam. Early and late changes in markers of aortic stiffness with breast cancer therapy. *Intern. Med. J.* 45(2):140–147, 2015. 10.1111/imj.12645 25404097

[23] Husmann, M., V. Jacomella, C. Thalhammer, and B. R. Amann-Vesti. Markers of arterial stiffness in peripheral arterial disease. *Vasa.* 44(5):341–348, 2015. 10.1024/0301-1526/a000452 26317253

[24] O’Rourke, M. F., A. Adji, and M. E. Safar. Structure and function of systemic arteries: reflections on the arterial pulse. *Am. J. Hypertens.* 31(8):934–940, 2018. 10.1093/ajh/hpy084 30016416

[25] Davies, J. I., and A. D. Struthers. Beyond blood pressure: pulse wave analysis–a better way of assessing cardiovascular risk? *Future. Cardiol.* 1(1):69–78, 2005. 10.1517/14796678.1.1.69 19804062

[26] Yilin, Z., F. Haiquan, H. Chen, and S. Juan. Hemodynamics of asymmetrically stenotic vertebral arteries based on fluid-solid coupling. *J Biol. Phys.* 51(1):10, 2025. 10.1007/s10867-025-09673-x PMC11833003 39961896

[27] Wang, W. Z., C. Liu, J. Q. Luo, L. J. Lei, M. H. Chen, Y. Y. Zhang, R. Sheng, Y. N. Li, L. Wang, X. H. Jiang, T. M. Xiao, Y. H. Zhang, S. W. Li, Y. X. Wu, Y. Xu, Y. N. Xu, and S. Y. Si. A novel small-molecule PCSK9 inhibitor E28362 ameliorates hyperlipidemia and atherosclerosis. *Acta. Pharmacol. Sin.* 45(10):2119–2133, 2024. 10.1038/s41401-024-01305-9 PMC11420243 38811775

[28] Hsiu, H., J. C. Liu, C. J. Yang, H. S. Chen, M. S. Wu, W. R. Hao, K. Y. Lee, C. J. Hu, Y. H. Wang, and Y. A. Fang. Discrimination of vascular aging using the arterial pulse spectrum and machine-learning analysis. *Microvasc. Res.* 139:104240, 2022. 10.1016/j.mvr.2021.104240 34508787

[29] Lin, S. K., H. Hsiu, H. S. Chen, and C. J. Yang. Classification of patients with Alzheimer’s disease using the arterial pulse spectrum and a multilayer-perceptron analysis. *Sci. Rep.* 11(1):8882, 2021. 10.1038/s41598-021-87903-7 PMC8076260 33903610

[30] Chen, C. C., C. K. Chang, C. C. Chiu, T. Y. Yang, W. R. Hao, C. H. Lin, Y. A. Fang, W. Jian, M. H. Hsu, T. L. Yang, J. C. Liu, and H. Hsiu. Machine learning analyses revealed distinct arterial pulse variability according to side effects of pfizer-BioNTech COVID-19 vaccine (BNT162b2). *J Clin. Med.* 11(20):6119, 2022. 10.3390/jcm11206119 PMC9605457 36294440

[31] Lin, Y. J., C. C. Lee, T. W. Huang, W. C. Hsu, L. W. Wu, C. C. Lin, and H. Hsiu. Using arterial pulse and laser doppler analyses to discriminate between the cardiovascular effects of different running levels. *Sens. (Basel).* 23(8):3855, 2023. 10.3390/s23083855 PMC10142346 37112196

[32] Wu, L. W., T. OuYoung, Y. C. Chiu, H. F. Hsieh, and H. Hsiu. Discrimination between possible sarcopenia and metabolic syndrome using the arterial pulse spectrum and machine-learning analysis. *Sci. Rep.* 12(1):21452, 2022. 10.1038/s41598-022-26074-5 PMC9744729 36509825

[33] Chen, C. T., H. Hsiu, S. H. Hung, G. Z. Chen, and Y. L. Huang. Characteristics of spectral indexes of the blood pressure waveform in patients with breast cancer. *Blood. Press. Monit.* 22(4):217–220, 2017. 10.1097/MBP.0000000000000260 28383291

[34] Chen, C. T., C. T. Ting, C. Y. Chen, Z. J. Lyu, C. C. Chen, Y. S. Chou, C. F. Cheng, C. H. Hsu, and H. Hsiu. Pulse-waveform and laser-doppler indices for identifying colorectal-cancer patients. *Biom. Eng. Appl. Basis. Comm.* 33(1):2150005, 2020.

[35] Chen, C. T., T. W. Huang, Y. S. Chou, C. F. Cheng, H. B. Wu, C. H. Hsu, and H. Hsiu. Effects of anthracycline chemotherapy and Kuan-Sin-Yin on the spectral indices of arterial pulse waveforms in breast cancer patients. *Clin. Hemorheol. Microcirc.* 84(4):345–358, 2023. 10.3233/CH-221602 37334580

[36] Hamet, P. Cancer and hypertension: a potential for crosstalk? *J. Hypertens.* 15(12 Pt 2):1573–1577, 1997. 10.1097/00004872-199715120-00058 9488208

[37] Yeo, W., F. K. F. Mo, E. Pang, J. J. S. Suen, J. Koh, H. H. F. Loong, C. C. H. Yip, et al. Profiles of lipids, blood pressure and weight changes among premenopausal chinese breast cancer patients after adjuvant chemotherapy. *BMC. Womens. Health.* 17(1):55, 2017. 10.1186/s12905-017-0409-8 PMC5532788 28750616

[38] Aviv, A., and H. Aviv. Reflections on telomeres, growth, aging, and essential hypertension. *Hypertens.* 29:1067–1072, 1997. 10.1161/01.hyp.29.5.1067 9149667

[39] Cohen, J. D., L. Li, Y. Wang, C. Thoburn, B. Afsari, L. Danilova, C. Douville, A. A. Javed, F. Wong, A. Mattox, R. H. Hruban, C. L. Wolfgang, M. G. Goggins, M. Dal Molin, T. L. Wang, R. Roden, A. P. Klein, J. Ptak, L. Dobbyn, J. Schaefer, N. Silliman, M. Popoli, J. T. Vogelstein, J. D. Browne, R. E. Schoen, R. E. Brand, J. Tie, P. Gibbs, H. L. Wong, A. S. Mansfield, J. Jen, S. M. Hanash, M. Falconi, P. J. Allen, S. Zhou, C. Bettegowda, L. A. Diaz Jr., C. Tomasetti, K. W. Kinzler, B. Vogelstein, A. M. Lennon, and N. Papadopoulos. Detection and localization of surgically resectable cancers with a multi-analyte blood test. *Science.* 359(6378):926–930, 2018. 10.1126/science.aar3247 PMC6080308 29348365

[40] Mikael, L. R., A. M. G. Paiva, M. M. Gomes, A. L. L. Sousa, P. C. B. V. Jardim, P. V. O. Vitorino, M. B. Euzébio, W. M. Sousa, and W. K. S. Barroso. Vascular aging and arterial stiffness. *Arq. Bras. Cardiol.* 109(3):253–258, 2017. 10.5935/abc.20170091 PMC5586233 28678931

[41] DuPont, J. J., R. M. Kenney, A. R. Patel, and I. Z. Jaffe. Sex differences in mechanisms of arterial stiffness. *Br J Pharmacol.* 176(21):4208–4225, 2019. 10.1111/bph.14624 PMC6877796 30767200

[42] Yerukala Sathipati, S., and S. Y. Ho. Identifying a miRNA signature for predicting the stage of breast cancer. *Sci. Rep.* 8(1):16138, 2018. 10.1038/s41598-018-34604-3 PMC6208346 30382159

[43] Athira, K., and G. Gopakumar. Breast cancer stage prediction: a computational approach guided by transcriptome analysis. *Mol. Genet. Genomics.* 297(6):1467–1479, 2022. 10.1007/s00438-022-01932-z 35922530

[44] Wang, Y., X. Liang, Z. Zhou, Z. Hou, J. Yang, Y. Gao, C. Yang, T. Chen, and C. Li. Prevalence and numbers of diabetes patients with elevated BMI in china: evidence from a nationally representative cross-sectional study. *Int. J. Environ. Res. Public. Health.* 19(5):2989, 2022. 10.3390/ijerph19052989 PMC8910421 35270682

[45] Zheng, M., X. Zhang, S. Chen, Y. Song, Q. Zhao, X. Gao, and S. Wu. Arterial stiffness preceding diabetes: a longitudinal study. *Circ. Res.* 127(12):1491–1498, 2020. 10.1161/CIRCRESAHA.120.317950 32985370

