# Supplementary material for: Discriminating Breast Cancer Patients by Spectral Analysis of Arterial Pulse Waveforms
Source: Cardiovasc Eng Technol. 2026 May 18;17(5):564–78. doi: 10.1007/s13239-026-00842-0 (PMC13723660; doi:10.1007/s13239-026-00842-0)
Supplement: Supplementary file 1 [file 13239_2026_842_MOESM1_ESM.doc]

The present analysis procedure included signal processing and information processing:

- signal processing

Frequency-domain analysis was applied to derive the 40 harmonic indices from the measured BPW signals (*n*=1-10): amplitude proportion (*C_n_*), coefficient of variation of *C_n_* (*CV_n_*), phase angle (*P_n_*), and standard deviation of *P_n_* (*P_n_*_*SD*).

Each individual pulse (between foot points) can be represented by the following finite series. The pulses were excluded if the values between the two foot points were larger than 20% of the pulse amplitude.

$$x(t)=\frac{A_{0}}{2}+\left\{\sum_{n=1}^{k/2} A_{n}\cos n\omega t_{s}+\sum_{n=1}^{k/2} B_{n}\cos n\omega t_{s}\right\}$$

The Fourier coefficients (*A_n_* and *B_n_*) of the pulse can be calculated as

$\begin{matrix} A_{n}=\frac{2}{k}\sum_{s=0}^{k} x_{s}\cos n\omega t_{s\text{ }}(for\text{ }n=0,1,...,\frac{k}{2}) \\ B_{n}=\frac{2}{k}\sum_{s=0}^{k} x_{s}\sin n\omega t_{s\text{ }}(for\text{ }n=0,1,...,\frac{k}{2}) \end{matrix}$

where $\omega$ is the angular frequency and $t_{s}$ is the sampling time interval.

The amplitude (*Amp_n_*) and phase angle (*P_n_*) of each harmonic of the pulse harmonic spectrum can then be calculated as $Amp_{n}=\sqrt{A_{n}^{2}+B_{n}^{2}}$ and $P_{n}=\arctan(B_{n}/A_{n})$. The amplitude proportions (*C_n_* values) for each pulse were calculated as *Amp_n_* / *Amp*_0_ × 100%, for *n* = 1–10. *CV*_n_ was then calculated as the coefficient of variations of *C_n_*, and *P*_n__*SD* was calculated as the standard deviation of *P*_n_.

- information processing

For information processing, the features of pulse signals were collected from the results of the signal-processing stage described above, to yield 40 indices for each pulse: *C_n_*, *CV_n_*, *P_n_*, and *P_n_*_*SD* values for *n* = 1–10. The two classification methods of ML analysis and PDA were used to discriminate the BC and Control groups. The confusion matrix was constructed, and the accuracy, sensitivity, specificity, and AUC were calculated to evaluate the performance of each ML classifier and PDA score. For the ML analysis, the following eight supervised methods were used for the binary classification of the data. Threefold cross-validation was used in the model training process.

Parameters of the machine-learning models.

| machine-learning methods | model parameters |
| --- | --- |
| SVM  (support vector machine) | C=1; kernel: rbf; gamma: auto; tol= 0.0001; max_iter=-1; class_weight: none |
| MLP  (multilayer perception) | hidden_layer_sizes=100; solver: adam; alpha=0.0001; batch_size: auto; max_iter=200; learning_rate_int=0.001 |
| GNB  (Gaussian Naive Bayes) | Priors: none |
| DT  (decision tree) | Criterion: gini; Splitter: best; max_depth: none; min_samples_split=2; min_samples_leaf=1; min_weight_fraction_leaf=0; max_features: none; max_leaf_nodes: none; min_impurity_split=0.0 |
| RF  (random forest) | n_estimators=100; criterion: gini; max_depth: none; min_samples_split=2; min_samples_leaf=1; min_weight_fraction_leaf=0; max_features: none; max_leaf_nodes: none |
| LR  (logistic regression) | Penalty: l2; Solver: lbfgs; multi_class: auto; class_weight: none |
| LDA  (linear discriminant analysis) | Solver: svd; Shrinkage: none; Priors: none |
| KNN  (K-nearest neighbor classification) | n_neighbors=5; weights: uniform; algorithm: auto; n_jobs: none; p: none |

For PDA, spectral indices that differed significantly between the BC and Control groups were used to calculate a score that illustrated the similarity of the pulse waveform with a Control or BC characterization. The PDA method is a feature scoring technique designed to work with a limited number of subjects. It doesn't create new features from the raw signal but instead provides a structured way to weight and combine pre-calculated spectral indices. The process can be broken down into four key steps:

1. Feature extraction (Preprocessing for PDA):

Before PDA begins, the raw arterial pulse waveform is processed to generate 40 standard spectral indices for each pulse. These are the features PDA will use amplitude proportions (*Cₙ* for n=1-10), variability of amplitude (*CVₙ*), phase angles (*Pₙ*) and variability of phase (*Pₙ_SD*).

2. Feature selection:

The PDA method is not applied to all 40 indices. Instead, it first performs statistical tests (e.g., t-tests) to identify which indices show either: significant differences (*p* < 0.05) or marginally significant differences (0.05 < *p* < 0.10) between the control group and the BC groups). Only these selected indices proceed to the scoring step.

3. Scoring mechanism (The core of PDA):

This is the discriminative step. For each selected index, a subject's value is converted into a score based on its position within the distribution of the control and BC groups. A value of 0 to 10 is assigned based on where the subject's value falls within these distributions. If the BC group tends to have higher values for an index, a subject's value is scored based on the control group's probability distribution function. A value deep within the control distribution gets a score near 0 ("very control-like"), while a value in the tail gets a score near 10 ("very BC -like"). Conversely, if the BC group tends to have lower values, scoring is based on the BC group's probability distribution function. A value deep within the BC distribution gets a score near 0, and a value in the control group's tail gets a score near 10. This creates a "PDA score" for each selected index, quantifying how "BC-like" that particular feature is for the subject.

4. Score aggregation and classification:

The final PDA Score for a subject is the average of the individual scores from all selected indices. A low average score indicates the subject's overall pulse spectrum is very similar to the healthy control characterization. A high average score indicates a strong resemblance to the BC characterization. A threshold is then chosen to classify subjects into "control" or "BC" groups based on this aggregated score.

PDA acts as a sophisticated, weighted voting system. It identifies which spectral features are most altered by the BC effects, translates the degree of alteration for each feature into a standardized score, and then averages these scores to produce a single, powerful discriminative metric.

The research group has previously developed and validated the PDA methodology in several other clinical contexts, confirming its reliability. For example, it has been demonstrated that PDA could effectively discriminate between patients with possible sarcopenia, metabolic syndrome, and healthy controls using arterial pulse signals, showcasing its application in muscular and metabolic disorders [32].
